# Molecular Phylogeny of Weakfish Species of the Stellifer Group (Sciaenidae, Perciformes) of the Western South Atlantic Based on Mitochondrial and Nuclear Data

**DOI:** 10.1371/journal.pone.0102250

**Published:** 2014-07-11

**Authors:** Andressa Jisely Barreto Barbosa, Iracilda Sampaio, Horacio Schneider, Simoni Santos

**Affiliations:** Federal University of Pará, Laboratory of Genetics and Molecular Biology, Institute of Coastal Studies - IECOS, Bragança, Pará, Brazil; Institute of Biochemistry and Biology, Germany

## Abstract

The phylogenetic relationships within the Stellifer group of weakfishes (*Stellifer*, *Odontoscion*, *Ophioscion*, and *Bairdiella*) were evaluated using 2723 base pairs comprising sequences of nuclear (rhodopsin, TMO-4C4, RAG-1) and mitochondrial (16S rRNA and COI) markers obtained from specimens of nine species. Our results indicate a close relationship between *Bairdiella* and Odontoscion, and also that the genus *Stellifer* is not monophyletic, but rather that it consists of two distinct lineages, one clade containing *S. microps/S. naso/S. brasiliensis* and the other, *S. rastrifer/S. stellifer/Stellifer* sp. B, which is closer to *Ophioscion* than the former clade. The *O. punctatissimus* populations from the northern and southern Brazilian coast were also highly divergent in both nuclear (0.8% for rhodopsin and 0.9% for RAG-1) and mitochondrial sequences (2.2% for 16S rRNA and 7.3% for COI), which we conclude is consistent with the presence of two distinct species. The morphological similarities of the members of the Stellifer group is reinforced by the molecular data from both the present study and previous analyses, which have questioned the taxonomic status of the Stellifer group. If, on the one hand, the group is in fact composed of four genera (*Stellifer*, *Ophioscion*, *Odontoscion*, and *Bairdiella*), one of the two *Stellifer* clades should be reclassified as a new genus. However, if the close relationship and the reduced genetic divergence found within the group is confirmed in a more extensive study, including representatives of additional taxa, this, together with the morphological evidence, would support downgrading the whole group to a single genus. Obviously, these contradictory findings reinforce the need for a more systematic taxonomic revision of the Stellifer group as a whole.

## Introduction

The family Sciaenidae includes approximately 70 genera and 270 species of demersal fishes found mainly over muddy or sandy bottoms of the continental shelf of the Atlantic, Indian, and Pacific oceans, as well as freshwater genera in the rivers of the Old and New Worlds [1], [2]. In the western South Atlantic, sciaenids are abundant and highly diverse, encompassing approximately 50 species representing 19 genera [3], [4].

Chao [5] evaluated the phylogenetic relationships of the 21 western Atlantic sciaenid genera and two freshwater genera based on morphological traits, and identified 11 suprageneric groups: *Micropogonias*, *Nebris*, *Pogonias*, *Sciaenops*, *Larimus*, *Sciaena*, *Umbrina*, *Menticirrhus*, *Lonchurus*, *Cynoscion*, and *Stellifer*. Of these groups, Stellifer can be distinguished from all the others by the presence of two (rather than one) pairs of large otoliths and a swim bladder with two (rather than one) chambers.

The Stellifer group includes four genera – Stellifer, Ophioscion, Bairdiella, and Odontoscion – represented by 12 species in the western South Atlantic: Stellifer naso, S. griseus, S. venezuelae, S. brasiliensis, S. microps, S. rastrifer, S. stellifer, Stellifer. sp. A, Stellifer. sp. B, Odontoscion dentex, Ophioscion punctatissimus, and Bairdiella ronchus [5]. These species are characterized by a very strong second anal spine, two pairs of large otoliths, and a swim bladder with two chambers, a carrot-shaped posterior chamber, and the anterior one yoke-shaped with a pair of diverticula on the posterolateral surface [4], [5].

Species of the Stellifer group are widely distributed in the western Atlantic, where they are abundant in coastal and estuarine waters with sandy or muddy bottoms [6], [7], including the coast of Brazil [8]–[15]. This group is especially appropriate for studies of the genetic connectivity of populations because the species are widely distributed, and normally inhabit estuarine environments. Despite this, few studies have focused on the bio-ecological or phylogenetic characteristics of this group. Regarding the phylogenetic relationships, all the available studies [1], [5], [16], [17] have emphasized the close relationships among *Bairdiella, Stellifer, Ophioscion*, and *Odontoscion*, although intergeneric and interspecific relationships have yet to be defined conclusively due to the limitations or inconsistencies found in the data, as described below.

The first phylogeny based on morphological traits was proposed by Chao [5], who concluded that *Stellifer* is most closely related to *Ophioscion*, with *Bairdiella* appearing as a sister group to *Odontoscion*. In a subsequent morphological study, Sasaki [1] suggested that *Ophioscion* and *Stellifer* are sister groups which form a clade with *Bairdiella*, whereas *Odontoscion* is related to the sciaenids of the eastern Pacific, *Elattarchus* and *Corvula*.

In a phylogenetic study based on 16S rRNA sequences, Vinson et al. [16] confirmed the close relationship between *Stellifer* and *Bairdiella*, although they did not include *Ophioscion* or *Odontoscion* in their analyses, impeding the systematic assessment of the evolutionary relationships within the group. In a recent study based on both mitochondrial (COI and 16S rRNA) and nuclear markers (TMO-4C4), Santos et al. [17] concluded that *Stellifer* is a sister group of *Ophioscion* and that *Bairdiella* is the basal taxon within the group, confirming the proposal of Sasaki [1]. However, as in Vinson et al. [16], the relationships between all of the taxa of the Stellifer group could not be defined because *Odontoscion* was not included in the analyses. Additionally, the relationships among the *Stellifer* species remain unclear, given that, in Vinson et al. [16], *S. microps* is a sister group to *S. naso* and *S. rastrifer* is closely related to *S. stellifer*, whereas in Santos et al. [17], *S. rastrifer* is a sister group to *Stellifer* sp., and *S. stellifer* is more closely related to *O. punctatissimus*.

In addition to the divergences in the conclusions of the morphological studies regarding the intergeneric relationships within Stellifer group, then, there are also disagreements among molecular phylogenies, especially with regard to the relationships among the *Stellifer* species. Given this, the present study evaluates the phylogenetic relationships within the Stellifer group, including all of its genera, using nuclear (TMO-4C4, RAG-1, and rhodopsin) and mitochondrial (16S rRNA and COI) markers, all of which have been widely used in phylogenetic reconstructions of fish taxa [17]–[27].

## Materials and Methods

### Ethics Statement

The species analyzed in the present study are not endangered or protected in the regions from which samples were obtained. The specimens were captured by artisanal fishers and processed (collection, handling, transportation, and DNA extraction) with the authorization of the Brazilian Environment Ministry through permit number 12773–1 emitted in the name of Dr. Iracilda Sampaio. All work was performed in compliance with and approved by the Ethics Committee of the Federal University of Pará.

### Sampling

A total of 36 samples representing nine species of the four genera of the Stellifer group distributed in the western South Atlantic were collected along the Brazilian coast (Table 1). Most of the specimens were obtained from the Sciaenidae tissue bank of the UFPA Genetics and Molecular Biology Laboratory of the Institute of Coastal Studies in Bragança, Brazil. The species were identified using the specialized literature [5], and muscle tissue was extracted from each specimen and conserved in absolute ethanol and frozen until analysis in the laboratory.

**Table 1:** Species and genomic regions used in the present study, including the samples used as outgroups.

Family	Species	*N*	Brazilian state of origin	GenBank accession number				
				16S rRNA	COI	TMO-4C4	RHOD	RAG-1
Sciaenidae	Ingroup							
	*Bairdiella ronchus*	2	Pará	JX903962, KJ907197	KJ907229, KJ907230	JX904028, KJ907267	KJ907299, KJ907300	KJ907335, KJ907336
	*Bairdiella ronchus*	2	São Paulo	KJ907198, KJ907199	KJ907231, KJ907232	KJ907268, KJ907269	KJ907301, KJ907302	KJ907337
	*Odontoscion dentex*	5	Espírito Santo	KJ907200–KJ907204	KJ907233–KJ907237	KJ907270–KJ907274	KJ907303–KJ907307	KJ907338–KJ907342
	*Ophioscion punctatissimus*	2	Pará	JX903981, KJ907205	KJ907238, KJ907239	JX904047, KJ907275	KJ907308, KJ907309	KJ907343, KJ907344
	*Ophioscion punctatissimus*	3	São Paulo	KJ907206–KJ907208	KJ907240–KJ907242	KJ907276–KJ907278	KJ907310–KJ907312	KJ907345, KJ907346
	*Stellifer brasiliensis*	3	São Paulo	JX903988, KJ907209, KJ907210	KJ907243–KJ907245	JX904054, KJ907279, KJ907280	KJ907313–KJ907315	KJ907347
	*Stellifer microps*	2	Pará	KJ907211, KJ907212	KJ907246, KJ907247	KJ907281, KJ907282	KJ907316, KJ907317	KJ907348
	*Stellifer naso*	3	Pará	KJ907213–KJ907215	KJ907248–KJ907250	KJ907283–KJ907285	KJ907318, KJ907319	-
	*Stellifer rastrifer*	4	Pará	KJ907216–KJ907219	KJ907251–KJ907254	KJ907286–KJ907289	KJ907320–KJ907323	KJ907349–KJ907352
	*Stellifer rastrifer*	1	Santa Catarina	KJ907220	KJ907255	KJ907290	KJ907324	KJ907353
	*Stellifer* sp. B	5	São Paulo	JX903992, KJ907221–KJ907223	KJ907256–KJ907260	JX904058, KJ907291–KJ907293	KJ907325–KJ907328	KJ907354–KJ907357
	*Stellifer stellifer*	3	Pará	JX903991, KJ907224, KJ907225	KJ907261–KJ907263	JX904057, KJ907294, KJ907295	KJ907329–KJ907331	KJ907358, KJ907359
	*Stellifer stellifer*	1	São Paulo	KJ907226	KJ907264	KJ907296	KJ907332	KJ907360
	Outgroup							
Lutjanidae	*Lutjanus purpureus*	1	-	KJ907227	KJ907265	KJ907297	KJ907333	KJ907361
	*Ocyurus chrysurus*	1	-	KJ907228	KJ907266	KJ907298	KJ907334	KJ907362

GenBank accession numbers are listed. N is the number of individuals used, and the Brazilian state of origin is the site where the samples were collected.

### DNA Extraction, PCR, and Genomic Sequencing

Total DNA was extracted by using the Wizard genomic DNA purification kit (Promega, Madison, Wisconsin, USA) following the protocol for extraction from muscle tissue as defined by the manufacturer. To evaluate the quality of the DNA, samples were electrophoresed in 1% agarose gel stained with GelRed (Biotium Inc., Hayward, California, USA) and analyzed under a UV transilluminator.

The mitochondrial (16S rRNA and COI) and nuclear (TMO-4C4, RAG-1, and rhodopsin) regions were amplified by PCR using the primers and amplification cycles described in Table 2. The RAG-1 region was amplified using a nested PCR, in which the primers 2510F [20] and RAG1R1 [32] were used first, followed by a second amplification using the primers RAG1F1 and RAG1R2 [32]. The reactions were conducted in a final volume of 25 µl, containing 4 µl of dNTPs (1.25 mM), 2.5 µl of PCR buffer (10X), 1 µl of MgCl_2_ (50 mM), 1 µl of DNA (100 ng/µl), 1 µl of each primer (50 ng/µl), 0.2 µl of Taq DNA Polymerase (5 U/µL, Invitrogen, Carlsbad, California, USA), and sterile water to complete the final volume. The PCR products were run on an agarose gel (1%) stained with GelRed (Biotium Inc., Hayward, California, USA) to verify the quality of the amplification products under ultraviolet light.

**Table 2:** Primers and amplification protocols for the mitochondrial and nuclear markers.

Marker	Primer	Reference	Amplification protocol
16S rRNA	L1987: 5′ GCCTCGCCTGTTTACCAAAAAC 3′	Modified from Palumbi [28]	Initial denaturation at 94°C for 3′; 30 cycles at 94°C for 20″(denaturation), 50°C for 30″(annealing), and 72°C for 30″; and final extension at 72°C for 3′
	H2609: 5′ CCGGTCTGAACTCAGATCACGT 3′		
COI	FishF1: 5′ TCAACCAACCACAAAGACATTGGCAC 3″	[29]	Initial denaturation at 94°C for 3′; 30 cycles at 94°C for 40″(denaturation), 59°C for 30″(annealing), and 72°C for 30″; and final extension at 72°C for 7′
	FishR1: 5′ TAGACTTCTGGGTGGCCAAAGAATCA 3′		
TMO-4C4	F2: 5′ CGGCCTTCCTAAAACCTCTCATTAAG 3′	[30]	Initial denaturation at 95°C for 2′; followed by 35 cycles at 95°C for 30″ (denaturation), 60°C for 30″(annealing), and 72°C for 1′; and final extension at 72°C for 7′
	R2: 5′ GTGCTCCTGGGTGACAAAGTCTACAG 3′		
Rhodopsin	Rod-F2 W: 5′ AGCAACTTCCGCTTCGGTGAGAA 3′	[31]	Initial denaturation at 95°C for 7′; 40 cycles at 94°C for 30″(denaturation), 59°C for 30″(annealing), and 72°C for 30″; and final extension at 72°C for 7′
	Rod-4R: 5′ CTGCTTGTTCATGCAGATGTAGAT 3′		
RAG-1	2510 L: 5′ TGGCCATCCGGGTMAACAC 3′	[20], [32]	Initial denaturation at 94°C for 3′; followed by 40 cycles at 94°C for 30″(denaturation), 58°C for 45″(annealing), and 72°C for 45″; and final extension at 72°C for 10′
	RAG1R1: 5′ CTGAGTCCTTGTGAGCTTCCATRAAYTT 3′		
RAG-1	RAG1F1: 5′ CTGAGCTGCAGTCAGTACCATAAGATGT 3′	[32]	Initial denaturation at 94°C for 3′; followed by 40 cycles at 94°C for 30″ (denaturation), 58°C for 45″ (annealing), and 72°C for 45″; and final extension at 72°C for 10′
	RAG1R2: 5′ TGAGCCTCCATGAACTTCTGAAGRTAYTT 3′		

The positive PCR products were purified with ExoSAP-IT (Affymetrix, Cleveland, Ohio, USA) following the manufacturer's instructions, and sequenced by the di-deoxyterminal method with reagents from the BigDye Terminator v3.1 Cycle Sequencing kit (Applied Biosystems, Foster City, California, USA). Electrophoresis was conducted in an ABI 3500XL automatic sequencer (Applied Biosystems).

### Phylogenetic and Nucleotide Divergence Analyses

The sequences obtained were manually edited, and aligned using the CLUSTAL W algorithm [33] implemented in the BioEdit 7.2.5 program [34]. Some of the 16S rRNA and TMO-4C4 sequences included in the analysis were obtained from GenBank (see Table 1). Nucleotide saturation of each set of data was evaluated by plotting transitions and transversions against genetic distances in DAMBE 4.0.65 [35].

Phylogenetic relationships were reconstructed based on both the individual data sets (per gene) and the concatenated data, using maximum parsimony, maximum likelihood, and normal and hierarchical Bayesian inference approaches. Two species of the family Lutjanidae, *Ocyurus chrysurus* and *Lutjanus purpureus*, the probable sister group of the Sciaenidae, were used as the outgroups for all analyses (Table 1). The evolutionary models used in the phylogenetic reconstructions were obtained in jModeltest 0.1.1 [36]. The maximum parsimony analysis was run using a heuristic search with 1,000 random step-wise additions, using the subtree pruning-regrafting (SPR) algorithm with branch-swapping in PAUP* 4.0b10 [37]. The maximum likelihood tree was constructed in PhyML v3.0 [38] using a heuristic search to find the most probable topologies based on the substitution models TIM2ef+I+G (for 16S rRNA), TIM2+I+G (COI), K80+I (TMO-4C4), TIM1+G (rhodopsin), and TrNef+I+G (RAG-1), and, TPM1uf+I+G for the concatenated data set. Statistical support for the maximum parsimony and likelihood analyses was determined using 1,000 bootstrap pseudoreplicates [39].

Bayesian inference analyses were run in MrBayes 3.1.2 [40] using the evolutionary models TPM2+G (for 16S rRNA), TrN+I+G (COI), K80+I (TMO-4C4), TPM1+G (rhodopsin), and K80+I+G (RAG-1). Metropolis-coupled Markov chain Monte Carlo (MCMCMC) sampling was conducted with two independent runs of 3,000,000 generations to estimate the posterior probabilities of the observed clades, using the parameters defined by the models as starting values. The Bayesian posterior probabilities for the clades were determined using the 50% consensus rule for trees sampled every 20 generations after removing the trees produced before the chains became stationary. The burn-in was empirically defined by evaluating the likelihood values. Convergence of the data was evaluated by verifying the parameters throughout the generations in Tracer 1.5 [41].

A species tree was constructed according to the hierarchical Bayesian inference principle in the BEAST 1.7.4 software package [42]. In this analysis, one tree was defined *a priori*, and each species of the group was considered to be a valid taxon. Markov chain Monte Carlo (MCMC) sampling was performed for 450 million generations with parameters sampled every 1,000 generations, and an initial burn-in of 10%. Convergence of the parameters was evaluated in Tracer 1.5 [41]. All of the trees obtained were viewed and edited in FigTree 1.4.0 [43].

Nucleotide divergence within and among the lineages for each set of data were assessed using uncorrected *p* distances in the MEGA 5.2.2 program [44].

## Results

A total of 2723 base pairs, including 432 bps for rhodopsin, 401 bps for TMO-4C4, and 752 bps for RAG-1, as well as 508 bps for the mitochondrial 16S rRNA and 630 bps for the COI were obtained from 26 of the 36 specimens analyzed. None of the markers was saturated (data not shown). The complete database of both nuclear and mitochondrial sequences includes 549 sites that are informative for parsimony analysis, with an overall transition/transversion ratio of 3.6.

**Figure 1:**
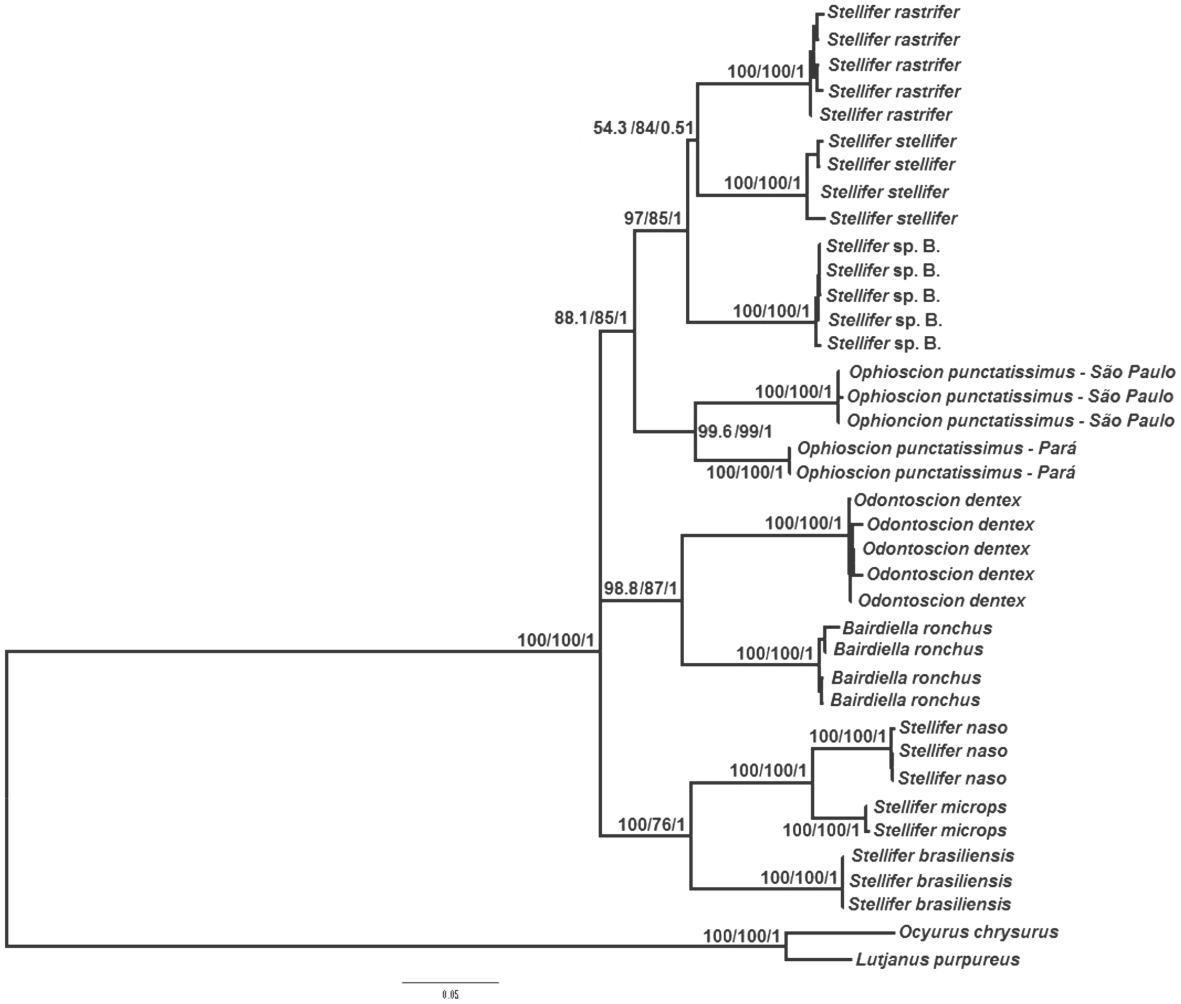
Maximum likelihood tree for the Stellifer group, based on mitochondrial (COI and 16S rRNA) and nuclear DNA sequences (rhodopsin, TMO-4C4, and RAG-1). The numbers above the branches represent the bootstrap values for maximum likelihood and maximum parsimony, and posterior Bayesian probabilities, respectively.

**Figure 2:**
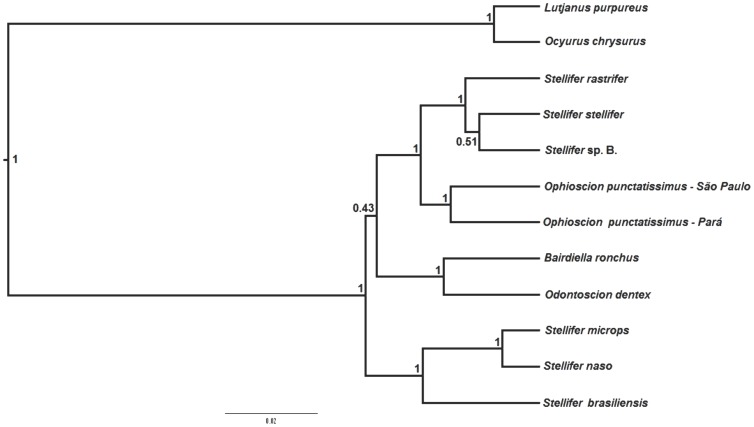
Species tree of the Stellifer group constructed from sequences of mitochondrial (COI and 16S rRNA) and nuclear DNA (rhodopsin, TMO-4C4, and RAG-1). The numbers above the branches indicate the posterior probabilities for the respective clade.

As the maximum parsimony, maximum likelihood, and Bayesian inference trees all presented similar topologies, only the maximum likelihood tree is shown here (Figure 1). The principal difference among the trees was in the position of *S. stellifer*, which grouped with *Stellifer* sp. B in the Bayesian species tree (Figure 2), but is the sister group of *S. rastrifer* in the other trees (Figure 1). In both cases, however, the statistical support is weak. All the results suggest the monophyly of the Stellifer group, with significant bootstrap and posterior probability values (Figures 1 and 2). However, it was not possible to determine which of the group's lineages is basal because all three approaches produced a polytomous arrangement (Figures 1 and 2).

The close relationship between *Bairdiella* and *Odontoscion* was well supported in all of the analyses (Figures 1 and 2). Our results also suggest that the genus *Stellifer* is not monophyletic because the species *S. rastrifer*, *S. stellifer*, and *Stellifer* sp. B form a clade closely related to *Ophioscion*, with significant statistical support (Figures 1 and 2), whereas *S. microps, S. naso*, and *S. brasiliensis* form a distinct clade, which is also strongly supported by bootstrap and posterior probability values (Figures 1 and 2).

Regarding the interspecific relationships within genus *Stellifer*, *S. naso* is a sister group to *S. microps*, composing a clade along with *S. brasiliensis* (Figures 1 and 2). In the second clade containing the other species of *Stellifer*, the low bootstrap and posterior probability values did not allow a reliable definition of the evolutionary relationships among *Stellifer* sp. B, *S. rastrifer* and *S. stellifer* (Figures 1 and 2).

All the analyses supported the separation of the northern (Pará) and southern (São Paulo) lineages of *O. punctatissimus*, based on high bootstrap and posterior probability values (Figures 1 and 2).

## Discussion

This is the first molecular phylogeny that includes species representative of all four genera of the Stellifer group, as proposed by Chao [5]. The results of all of the analyses suggest the monophyly of the group (Figures 1 and 2), and are consistent with those of morphological analyses [5] and a molecular study of 17 sciaenid genera, including those of the Stellifer group [17]. However, as the Sciaenidae is a large family that includes some 70 genera, further analyses including the Stellifer group and other closely-related sciaenids, will be necessary for a more conclusive evaluation of the group's monophyletic status.

*Bairdiella* is a sister group to *Odontoscion* in all the topologies generated in the present study (Figures 1 and 2), which corroborate Chao's [5] arrangement, based on morphological traits. By contrast, the findings of Sasaki [1] indicate that *Stellifer*/*Ophioscion/Bairdiella* share a common ancestor, whereas *Odontoscion* would be more closely related to the eastern Pacific *Ellatarchus* and *Corvulla*. These results contrast with those obtained in the present study and the phylogenies determined by Chao [5] and Santos et al. [17]. However, *Ellatarchus* and *Corvulla* were not included in either the present study or the previous ones [5], [17], which means that further phylogenetic analyses will be necessary to resolve these contradictions.

The results of the present study confirm that *Stellifer* is not monophyletic. The *Stellifer* sp. B/*S. rastrifer/S. stellifer* clade shares a common ancestry with *O. punctatissimus*, whereas *S. microps*, *S. naso*, and *S. brasiliensis* form a distinct clade, in both cases supported by significant bootstrap and posterior probability values (Figures 1 and 2). These results refute the morphology-based hypotheses [1], [5] and are consistent with the arrangement proposed by Santos et al. [17], who concluded that *Stellifer* comprises two distinct lineages, and that *Stellifer* sp. B/*S. stellifer/S. rastrifer* would be closer to *O. punctatissimus* than the second clade. Given these findings, we suggest that either that one of the two *Stellifer* clades should be assigned to a new genus or that the entire group should be subsumed into a single genus. Either way, additional morphological and molecular studies, including more species from the Stellifer group, will be necessary to reach a more conclusive evaluation of the phylogenetic relationship of this group.

Within *Stellifer*, our results corroborate the close phylogenetic relationship between *S. microps* and *S. naso* proposed by Vinson et al. [16], as well as the conclusions of Santos et al. [17] on the *S. naso/S. microps/S. brasiliensis* clade. However, our findings contrast with those of the latter study [17] with regard to the relationship between *S. rastrifer*, *S. stellifer*, and *Stellifer* sp. B. In the earlier study, *S. stellifer* was identified as a sister group of *O. punctatissimus*, whereas in the present one, this species is closer to its congeners than *Ophioscion* (Figures 1 and 2).

One surprising result of this study was the formation of two distinct and statistically well-supported clades of *O. punctatissimus* from northern (Pará) and southern (São Paulo) coasts of Brazil (Figure 1). In fact, genetic divergence in both mitochondrial and nuclear genes (2.2% for rRNA 16S, 7.3% for COI, 0.8% for TMO-4C4, 0.2% for Rhod, and 0.9% for RAG-1) is similar to or greater than that found between valid sciaenid species [16] and those of other fish families [23], [45], [46], which leads us to suggest that speciation occurred in the taxa. *Ophioscion punctatissimus* is the only species of this genus found in Brazil, which eliminates possible errors of identification of the specimens. The northern and southern populations are separated by more than 5000 km of coastline, and inhabitat areas with distinct geomorphological and oceanographic characteristics [47], [48], all of which may have contributed to a reduction in the gene flow between the two populations, and the differentiation observed in the present study.

A number of studies have nevertheless pointed out other factors, such as life-history traits, the ecological requirements of the species [49]–[53], or historic events, such as glaciations, as the primary determinants of genetic differentiation and speciation in fish [45], [54]–[57]. Population differentiation and speciation have been recorded in western Atlantic sciaenids, such as *Macrodon* [58], [59], which has two highly divergent lineages distributed in the western South Atlantic that were recently differentiated as *M. ancylodon* and *M. atricauda* by Carvalho-Filho et al. [60]. Mitochondrial and nuclear DNA sequences also indicate that the two distinct lineages of *Larimus breviceps* from the western South Atlantic may also represent distinct species [17], [61]. Given these findings, there is a clear need for more comprehensive data on the populations of *O. punctatissimus*, including additional molecular markers and specimens from a wider geographical area, in order to determine the exact levels of genetic differentiation and the range of each lineage.

In summary, the morphological similarities of the members of the Stellifer group [5] is reinforced by the molecular data from both the present study and previous analyses [16], [17], which have questioned the taxonomic status of the Stellifer group. If, on the one hand, the group is in fact composed of four genera (*Stellifer*, *Ophioscion*, *Odontoscion*, and *Bairdiella*), one of the two *Stellifer* clades should be reclassified as a new genus. However, if the close relationship and the reduced genetic diversity (data not shown) found within the group is confirmed in a more extensive study, including representatives of additional taxa, this, together with the morphological evidence, would support downgrading the whole group to a single genus. Obviously, these contradictory findings reinforce the need for a more systematic taxonomic revision of the Stellifer group as a whole.

## Conclusions

This study presents the most comprehensive molecular phylogeny yet produced for the genera of the Stellifer weakfish group. The analyses found close relationships among the taxa of the group, as well as two distinct lineages of *Stellifer*. In addition, marked genetic differentiation was found between the *O. punctatissimus* populations from northern and southern Brazil, suggesting that speciation occurred in the taxa. All these findings reinforce the need for more comprehensive analyses using both molecular markers and morphological traits for the definition of the phylogenetic relationships within the group.
